# Research on Capacitated Multi-Ship Replenishment Path Planning Problem Based on the Synergistic Hybrid Optimization Algorithm

**DOI:** 10.3390/biomimetics10050285

**Published:** 2025-05-02

**Authors:** Lin Yang, Qinghua Chen, Junjie Mu, Tangying Liu, Xiaoxiao Li, Shuxiang Cai

**Affiliations:** 1 Navy Aviation University, Yantai 264001, China; ylnaau304@163.com (L.Y.); a75145193@163.com (J.M.); lixiaoxiao8906@163.com (X.L.); 2 School of Electromechanical and Automotive Engineering, Yantai University, Yantai 264005, China; lty@ytu.edu.cn (T.L.); caisx8411@ytu.edu.cn (S.C.)

**Keywords:** path planning problem, capacitated multi-ship replenishment, synergistic hybrid optimization algorithm, ant colony algorithm, Clarke–Wright algorithm, genetic algorithm

## Abstract

Ship replenishment path planning is a critical problem in the field of maritime logistics. This study proposes a novel synergistic hybrid optimization algorithm (SHOA) that effectively integrates ant colony optimization (ACO), the Clarke–Wright algorithm (CW), and the genetic algorithm (GA) to solve the capacitated multi-ship replenishment path planning problem (CMSRPPP). The proposed methodology employs a three-stage optimization framework: (1) initial path generation via parallel execution of the CW and ACO; (2) population initialization for the GA by strategically combining optimal solutions from ACO and the CW with randomized solutions; (3) iterative refinement using an enhanced GA featuring an embedded evolutionary reversal operation for local intensification. To evaluate performance, the SHOA is benchmarked against ACO, the GA, the particle swarm optimization algorithm, and the simulated annealing algorithm for the capacitated vehicle routing problem. Finally, the SHOA is applied to diverse CMSRPPP instances, demonstrating high adaptability, robust planning capabilities, and promising practical potential.

## 1. Introduction

Ship replenishment path planning plays a crucial role in maritime transportation, representing a persistent and complex challenge in maritime logistics [1,2,3,4]. In practice, this task involves numerous intricate factors, including the quantity of supply vessels, capacity limitations, and the spatial distribution of supply points, which collectively contribute to the complexity of the route planning problem. The capacitated multi-ship replenishment path planning problem (CMSRPPP) seeks to identify the most efficient replenishment routes for a fleet of ships while respecting their capacity constraints, with the goal of minimizing the total travel distance or cost. This problem is essentially similar to the capacitated vehicle routing problem (CVRP) [5,6,7] and can be viewed as a resource allocation and route optimization challenge under specific constraints.

This type of problem can be solved by exact algorithms and heuristic methods [8,9,10]. Exact algorithms are based on rigorous mathematical principles. They establish mathematical models of the problem and employ mathematical techniques to solve it. Exact algorithms include the branch and bound algorithm [11], branch and cut algorithm [12], and dynamic programming [13], among others. While exact methods are capable of identifying the global optimal solution, their reliance on rigorous mathematical frameworks results in exponentially increasing computation times as the problem scale expands. For large-scale problems, it is often difficult to obtain results within a practically acceptable time, making exact algorithms only suitable for solving small-to medium-scale problems.

In comparison to exact algorithms, heuristic methods, though not ensuring the optimal solution, can yield satisfactory results within a limited timeframe. Heuristic methods can be categorized into traditional heuristic approaches and meta-heuristic strategies. Traditional heuristic approaches mainly include the Clarke–Wright algorithm (CW) [14] and the scanning method [15], among others. These traditional heuristics perform well on large-scale problems and offer fast solution speeds. However, they may be constrained by the search space when addressing complex problems. Meta-heuristic strategies, in contrast, demonstrate superior global search capabilities. Common meta-heuristic methods include ant colony optimization (ACO) [16,17], the genetic algorithm (GA) [18,19], the particle swarm optimization algorithm (PSO) [20], the simulated annealing algorithm (SA) [21], and the artificial bee colony algorithm [22], among others. Meta-heuristic methods possess robust global search capabilities, effectively avoid local optima, and demonstrate broad adaptability and strong adjustability. Their performance and solution quality can be optimized through parameter tuning.

However, since CVRP-like problems belong to NP-hard problems [23,24], their complexity increases with the problem scale. Traditional heuristic algorithms have not fully addressed the challenges of solving these problems effectively. Therefore, researchers continually advance heuristic methods and seek out efficient techniques to identify satisfactory solutions in a timely manner. Specific improvement measures include locally enhancing traditional heuristic algorithms or combining different meta-heuristic algorithms to design hybrid heuristic algorithms [25,26,27,28,29,30]. For example, Jin et al. [31] introduced a collaborative parallel heuristic method aimed at large-scale CVRP challenges. Ilhan [32] proposed a population-based SA for CVRP. Al-Tabeeb et al. [33] developed a hybrid firefly algorithm CVRP-FA that incorporates 2h-opt and modified 2-opt algorithms to enhance solution quality and hasten convergence. AKPINAR and Sener [34] introduced a novel hybrid large neighborhood ACO algorithm for CVRP solutions.

Therefore, enhancing heuristic methods to achieve satisfactory CVRP solutions within a reasonable computation time remains a crucial research focus. Among various heuristic approaches, the GA and ACO are widely utilized bio-inspired optimization techniques for addressing CVRP-like problems. The GA iteratively searches for optimal solutions by designing selection, crossover, and mutation operators. Although the traditional GA have global search capabilities, they are prone to premature convergence during the search process [35]. ACO is a swarm-based bio-inspired optimization algorithm with a positive feedback mechanism, strong robustness, and excellent local optimization ability. However, it requires long search times and may fall into local optima due to pheromone accumulation [36]. The above-mentioned characteristics indicate that the advantages of coordinating the two algorithms may break through the limitations of a single method.

According to the respective advantages of the GA and ACO, this study presents an innovative hybrid optimization approach. The algorithm first utilizes ACO and the CW to generate high-quality initial solutions. Subsequently, the optimal solutions from ACO and the CW and some random solutions are cleverly mixed to construct the initial population of genetic algorithm. During the GA iterations, an evolutionary reversal operation is embedded to adjust the order of path nodes, refining the solution locally and further enhancing its quality. The proposed hybrid optimization approach implements a hierarchical three-phase strategy: (1) high-quality initial solution generation through coordinated ACO and CW execution, (2) solution space diversification via hybrid population mechanisms, and (3) precision refinement employing an enhanced genetic algorithm. This architecture achieves optimal exploration–exploitation balance by orchestrating complementary interactions between the constituent algorithms’ strengths. The improved algorithm can not only provide high-quality solutions but also shows good performance in large-scale problem instances.

The key contributions of this study are as follows:

(1) A novel synergistic hybrid optimization algorithm (SHOA) integrating ACO, the GA, and the CW method is proposed to address the CMSRPPP.

(2) In the CVRP and the CMSRPPP, this method exhibits remarkable advantages in both solution quality and stability.

The structure of this paper is outlined as follows: Section 2 presents the capacitated multi-ship replenishment path planning problem; Section 3 outlines the proposed SHOA method; Section 4 assesses the performance of the proposed approach in solving the multi-ship replenishment route planning problem with capacity constraints; finally, Section 5 reviews the findings of this research and discusses potential future research directions.

## 2. Model for Ship Replenishment Path Planning Problem

This study focuses on an optimized maritime supply routing problem characterized by multi-vessel collaborative optimization under complex constraints. The challenge involves designing an optimal navigation scheme where multiple supply ships depart from the supply center, traverse all supply points, and then return to the same supply center. The routes of multiple supply ships cannot overlap, and the total transportation cost must be minimized. Additionally, the supply process must account for ship capacity constraints and risks caused by environmental factors that may alter the route. This issue parallels the common vehicle routing issue with constraints on load capacity.

Given the threats during the supply navigation process, this paper introduces a risk factor into the route planning process. This risk factor is then integrated into the route distance for subsequent planning. To prevent the counterintuitive scenario where risk-weighted distances (path lengths incorporating danger probabilities) might appear shorter than actual geometric distances, we define the risk factor *R_i_* for supply point *i* (where *P_i_* denotes the probability of encountering danger at that point) as follows:

(1) $$R_{i}=1+P_{i}$$

Consider a directed graph *G* = (*V*, *A*), where *V* is the set of nodes and *V* = {0, 1, 2,…, *n*}. Node 0 represents the supply center, while the remaining nodes *N* in the node set *V* represent the set of supply points, *N* = {1, 2,…, *n*}. A is the set of arcs, the supply center is equipped with *M* homogeneous ships, each with a capacity of *C*. The straight-line distance of arc (*i*, *j*) is *c_ij_*, and the cargo capacity of supply point *i* is *d_i_*. A feasible delivery route on the directed graph *G* must start and end at the supply center 0. In addition, ${∆}^{+}\left(i\right)$ represents the set of arcs departing from node *i*; ${∆}_{-}\left(j\right)$ represents the set of arcs returning to node *j*; and *K* denotes the set of delivery vehicles.

To facilitate modeling, the concepts of shortest straight-line distance and shortest risk distance are introduced. The shortest straight-line distance refers to the total straight-line distance of the route, disregarding the risk weights of sea areas. Its formula is as follows:

(2) $$\min\sum_{k\in K(i,j)\in A}c_{ij}x_{ijk}$$

where *c_ij_* indicates the distance between supply points *i* and *j* and *x_ijk_* indicates whether ship *k* departs from supply point *i* to supply point *j*. If so, *x_ijk_* = 1; otherwise, *x_ijk_* = 0.

The shortest risk distance accounts for both the straight-line distance of the route and the comprehensive impact of risk areas as the vessel navigates through them. To optimize algorithmic efficiency, this study adopts an edge-agnostic risk modeling framework. The composite risk metric for the inter-nodal path (*i*→*j*) is computationally derived from the product of constituent node risks as *R_i_* × *R_j_*, where *R_i_* is the risk factor of replenishment point *i*, and *R_j_* is the risk factor of replenishment point *j*. The formula is as follows:

(3) $$\min\sum_{k\in K(i,j)\in A}c_{ij}x_{ijk}R_{i}R_{j}$$

In summary, the mathematical model for the CMSRPPP is as follows:

(4) $$\min\sum_{k\in K(i,j)\in A}c_{ij}x_{ijk}R_{i}R_{j}$$

(5) $$\sum_{k\in K_{j}\in \Delta^{+}(i)}x_{ijk}=1\;\forall i\in N$$

(6) $$\sum_{j\in \Delta^{+}(0)}x_{0jk}=1\;\forall k\in K$$

(7) $$\sum_{i\in \Delta_{-}(j)}x_{ijk}-\sum_{i\in \Delta^{+}(j)}x_{jik}=0\;\forall k\in K,\forall j\in N$$

(8) $$\sum_{i\in \Delta^{-}(n+1)}x_{i,n+1,k}=1\;\forall k\in K$$

(9) $$\sum_{i\in N}d_{i}\sum_{j\in \Delta^{+}(i)}x_{ijk}⩽C\;\forall k\in K$$

(10) $$\sum_{i\in S}\sum_{j\notin S}x_{ijk}⩽|S|-1\;\forall S\subseteq N,|S|⩾2,\forall k\in K$$

(11) $$x_{ijk}\in \left\{\begin{array}{c} 0,1 \end{array}\right\}\;\forall k\in K,\forall (i,j)\in A$$

Equation (4) minimizes the total distance traveled by the ships for replenishment. Equation (5) ensures that each supply point is assigned to only one path. Equation (6) requires that ship *k* departs from the supply center exactly once. Equation (7) ensures that for each ship *k* and each supply point *j* (excluding the supply center), the flow entering node *j* equals the flow leaving node *j*. This balances the cargo flow at each node, meaning the amount of cargo delivered equals the amount removed. Equation (8) requires that ship *k* returns to the supply center once after replenishment. Equation (9) ensures that the post-replenishment load of ship *k* does not exceed the distribution ship’s maximum capacity. Equation (10) eliminates sub-loops that do not pass through the distribution center, where set *S* is a subset of set *N*. Equation (11) indicates whether ship *k* departs from supply point *i* to supply point *j*. If so, *x_ijk_* = 1; otherwise, *x_ijk_* = 0.

## 3. The Synergistic Hybrid Optimization Algorithm

### 3.1. Ant Colony Optimization

ACO [37], inspired by the behavior of ants as they forage, is a method where ants leave pheromone trails on their traversal paths. Subsequent ants are more likely to choose paths with a higher concentration of pheromones. The level of pheromone is influenced by both the number of ants that have traveled the path and its length; longer paths result in a lower pheromone concentration. Over time, ants prefer the path with the highest pheromone concentration. This method is highly effective for finding optimal routes.

The ACO workflow involves the following:

(1) Initialize the number of ants *m*, the number of nodes *n*, the pheromone importance factor *α*, the heuristic importance factor *β*, the pheromone evaporation factor *ρ*, and the pheromone matrix.

(2) Each ant starts from a randomly selected node in parallel.

(3) The next node is selected based on pheromone concentration and heuristic information. The probability that ant *k* moves from node *i* to node *j* is given by the formula:

(12) $$P_{ij}^{k}(t)=\left\{\begin{array}{ll} \frac{{\left[\tau_{ij}(t)\right]}^{\alpha }{\left[\eta_{ij}(t)\right]}^{\beta }}{\sum_{s\in allowed_{k}}{\left[\tau_{ij}(t)\right]}^{\alpha }{\left[\eta_{ij}(t)\right]}^{\beta }}, & j\in allowed_{k}\\ 0, & \mathrm{otherwise} \end{array}\right.$$

where *τ_ij_*(*t*) is the pheromone concentration at time *t* from node *j* to node *i*; *allowed_k_* is the set of possible next nodes for ant *k* at node *j*; *η_ij_*(*t*) is the heuristic information at time *t* from node *j* to node *i*; and its formula is as follows:

(13) $$\eta_{ij}(t)=\frac{1}{d_{ij}}$$

where *d_ij_* is the Euclidean distance from node *j* to node *i*.

(4) After all ants have completed a cycle, perform a global pheromone update:

(14) $$\tau_{ij}\left(t+1\right)=(1-\rho )\tau_{ij}\left(t\right)+\rho \Delta \tau_{ij}\left(t\right)$$

(15) $$\Delta \tau_{ij}\left(t\right)=\sum_{k=1}^{m}\Delta \tau_{ij}^{k}\left(t\right)$$

(16) $$\Delta \tau_{ij}^{k}(t)=\left\{\begin{array}{c} \frac{Q}{L_{k}}\\ 0 \end{array}\right.$$

*Q* is the initial value of pheromone intensity, and *L_k_* is the total length of the path taken by ant k.

### 3.2. Genetic Algorithm

The GA [38] is an optimization method that simulates the evolutionary processes of biological systems, characterized by strong problem-solving capabilities and broad adaptability. The GA procedure includes encoding the problem’s parameters, creating a random initial population, evaluating the fitness of each member, and then applying selection, crossover, and mutation. Once a predefined convergence criterion is achieved, the algorithm delivers the best solution found. If the criterion is not met, these operations are repeatedly applied to successive generations until the desired outcome is attained.

The implementation process of the GA includes the following steps:

(1) Initialization: randomly generate an initial population containing *N* individuals.

(2) Fitness Evaluation: calculate the fitness *f*(*x_i_*) for each individual *x_i_*.

(3) Selection: select individuals for reproduction based on their fitness.

(4) Crossover: randomly select two parents from the chosen individuals and perform crossover with probability *p_c_* to generate offspring.

(5) Mutation: apply random changes to the offspring with a small probability *p_m_.*

(6) Termination: repeat steps 2–6 until termination conditions are met, such as reaching the maximum number of generations or fitness convergence.

### 3.3. Clarke–Wright Algorithm

The CW [39] is a traditional heuristic approach employed to address the vehicle routing issue. Its primary function is to refine the paths of vehicle deliveries, aiming to minimize overall travel distances or expenses. The basic idea is to iteratively merge routes that yield the greatest distance savings.

(1) Initialization: Assign a separate route to each customer, meaning each vehicle serves only one customer.

(2) Calculate Savings: For each pair of customers *i* and *j*, calculate the savings achieved by merging their routes. The savings value is computed as follows:

(17) $$S_{ij}=d_{0i}+d_{0j}-d_{ij}$$

where *d*_0*i*_ and *d*_0*j*_ represent the distances from the depot (starting point) to customers *i* and *j*, respectively, and *d_ij_* is the distance between customers *i* and *j*.

(3) Sort Savings: Sort all savings values in descending order.

(4) Merge Routes: Starting with the pair of routes that offers the highest savings, attempt to merge them. If the merged route satisfies constraints such as vehicle capacity and time windows, execute the merge.

(5) Iterate: Repeat the above steps until no further merges are possible.

The Savings Algorithm is valued for its simplicity and efficiency, particularly for small- to medium-sized vehicle routing problems. However, it may not guarantee a global optimum due to its greedy strategy, which can lead to local optima.

### 3.4. An Evolutionary Reversal Operation

The reversal operation as a local search strategy, aiming to enhance the population’s exploration ability through structured perturbations while achieving local optimization combined with fitness evaluations (see Algorithm 1). The core idea of the reversal operation is to pick two indices *i* and *j* in an individual’s chromosome to reverse the sequence of elements in the gene segment between *i* and *j*, thereby generating a new gene combination. This operation enhances population diversity, prevents the algorithm from converging to local optima, and improves local search capabilities. To improve the local search capability of the GA, evolutionary reversal operation [40] is introduced after selection, crossover, and mutation. Evolutionary means that only those whose fitness values have improved after reversal are accepted, otherwise reversal is invalid.
**Algorithm 1:** Evolutionary reversal operation**Input**: A set of chromosomes to be optimized **Output**: Optimized population after evolutionary reversal operation **Begin**For each chromosome *C_k_* **in** the candidate population **do** Generate random indices *i*, *j* with 1 < *i* ≤ *j* < the chromosome’s column number Define the segment *S = C_k_ [i..j]* Reverse the segment: *S′ =* Reverse *(S)* Construct new chromosome *C_new_* Calculate original fitness *F_orig_ =* Fitness *(C_k_)* Calculate new fitness *F_new_ =* Fitness *(C_new_)* **If** *F*_new_ > *F*_orig_ **then**  Update chromosome*: C_k_←C_new_* **End If****End For**Integrate updated chromosomes into the optimized populationOutput optimized population**End**

### 3.5. A Novel Synergistic Hybrid Optimization Algorithm

The SHOA implements a three-phase hierarchical framework (see Algorithm 2):

(1) Parallelized Initialization: concurrent execution of ACO and CW generates diversified high-quality paths.

(2) Intelligent Population Construction: merge the ACO optimal solution, CW optimal solution, and random solution to form a high-quality initial population for genetic algorithm.

(3) Evolutionary Enhancement: iterative refinement using an enhanced GA featuring an embedded evolutionary re-versal operation for local intensification.

The three-phase framework achieves an ideal optimization trajectory: from initial high-quality solution generation (ACO+CW), through intermediate diversity preservation (hybrid population), to final refinement (enhanced GA), thereby establishing an optimal balance between exploration and exploitation while effectively combining ACO, the CW, and the GA.

**Figure 1:**
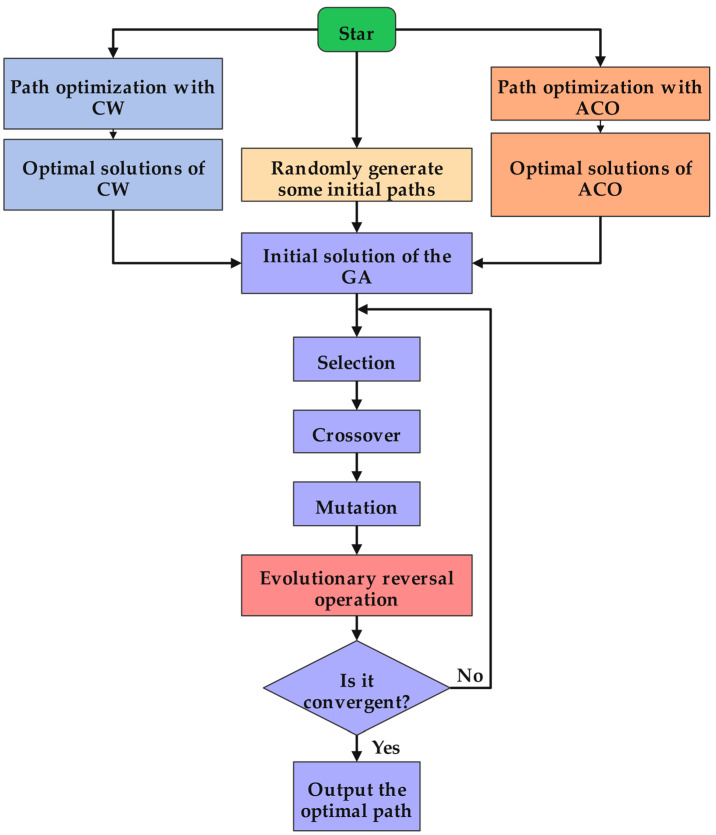
The flow chart of the SHOA.

The specific implementation steps of the improved algorithm are illustrated in Figure 1.
**Algorithm 2:** Synergistic Hybrid Optimization Algorithm **Input**: Ship replenishment nodes, vehicle capacity constraints **Output**: Optimized multi-ship replenishment paths **Begin** **Phase 1: Parallelized Initialization** ** Execute ACO in parallel:**Initialize parameters (*m*, *α*, *β*, *ρ*, pheromone matrix)Generate candidate paths via ACO’s probabilistic node selection and pheromone update rules **Execute CW in parallel:**Assign a route per replenishment nodesCompute savings, merge routes under capacity constraintsCollect optimal path *P_ACO_* obtained by ACO and optimal path *P_CW_* obtained by CW**Phase 2: Intelligent Population Construction**4.Generate *N*-2 random solutions *P_rand_* (*N* is the population size of the GA)5.Combine *P_ACO_*, *P_CW_*, and *P_rand_* to form initial GA population *P_initial_***Phase 3: Evolutionary Enhancement**6.While termination criteria not met do7. Evaluate fitness for each individual8. Record the current best solution9. Select parents via fitness-based selection10.  Perform crossover on parents with probability *p_c_*11.  Apply mutation to offspring with probability *p_m_*12.  Evolutionary reversal operation13.End WhileReturn the best solution **End**

## 4. Application of the Synergistic Hybrid Optimization Algorithm

In this study, we selected ACO, the GA, Hybrid Particle Swarm Optimization (HPSO), and the SA as comparison benchmarks primarily due to their proven effectiveness in path planning problems and combinatorial optimization. Specifically, ACO is a classic swarm intelligence algorithm often used for path optimization problems such as the Traveling Salesman Problem. The GA is a highly generalizable evolutionary algorithm suitable for solving various complex constrained optimization problems. HPSO used in this paper combines the social learning framework of PSO with deterministic genetic operators, including mandatory PMX crossover [41] and forced swap mutation [42]. The SA, which simulates the physical annealing process, helps solutions escape local optima and has been well-validated in the field of combinatorial optimization.

The parameter settings for all algorithms were carefully selected based on established practices in the literature and validated through preliminary experiments. For the ACO component, we set the pheromone importance factor (*α*) to 1, the heuristic importance factor (*β*) to 5, and the pheromone evaporation rate (*ρ*) to 0.1. The GA parameters included a selection probability of 0.9, crossover probability of 0.9, and mutation probability of 0.05, balancing exploration and exploitation. The SA was configured with an initial temperature of 1000 and cooling rate of 0.9 to ensure gradual convergence. Notably, the ACO and GA components within our proposed SHOA maintain identical parameter configurations to their standalone versions as specified above, ensuring consistent performance across hybrid implementations. All algorithms were run on a hardware platform consisting of an AMD Ryzen 7 4800H with Radeon Graphics with NVIDIA GeForce GTX 1650 Ti. The operating system used was Windows 10 (64-bit) with 16 GB of RAM.

### 4.1. Application of the Synergistic Hybrid Optimization Algorithm in the Capacitated Vehicle Routing Problem

The CMSRPPP discussed in this paper is similar to the CVRP. To assess the performance of the SHOA, we conducted tests on six CVRP instances with varying scales (10, 30, 60, 70, 90, and 100 customers). The depot coordinates were fixed at [0, 0], while customer coordinates were randomly generated as integers. Each customer’s demand was randomly assigned an integer value in increments of 100 within the range of 100–2500. These six CVRP instances were solved using the ACO, GA, HPSO, and SA methods, and the outcomes were contrasted with those of the SHOA. To ensure statistical reliability, all algorithms were executed 30 times.

**Figure 2:**
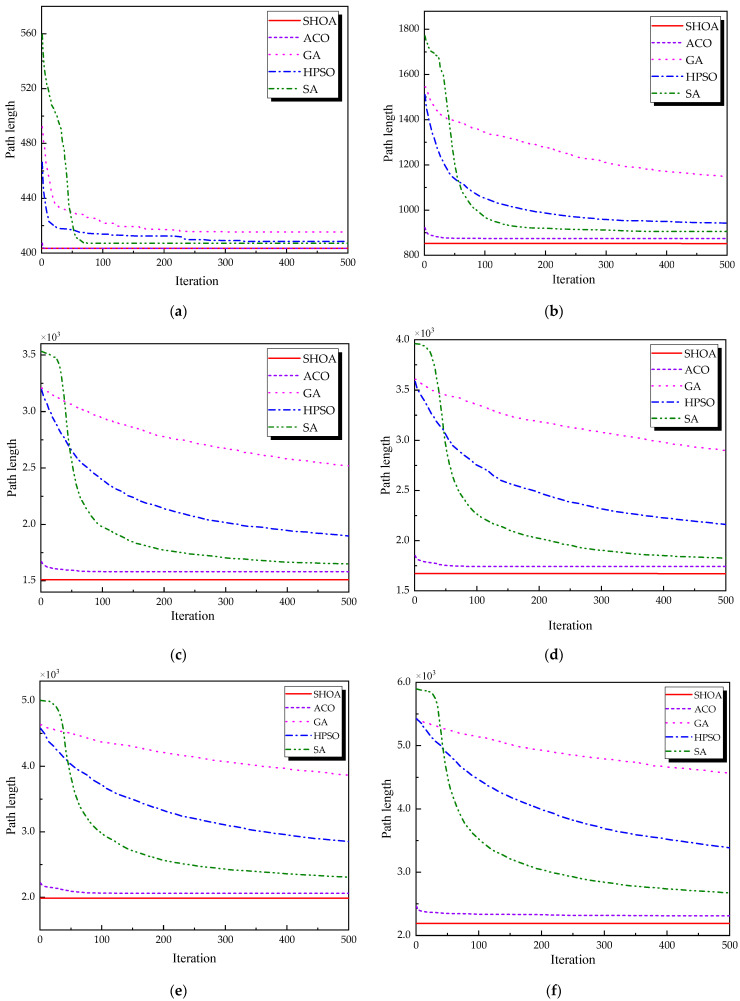
The average convergence curves derived from 30 runs of different algorithms. (**a**) 10 customers; (**b**) 30 customers; (**c**) 60 customers; (**d**) 70 customers; (**e**) 90 customers; (**f**) 100 customers.

**Figure 3:**
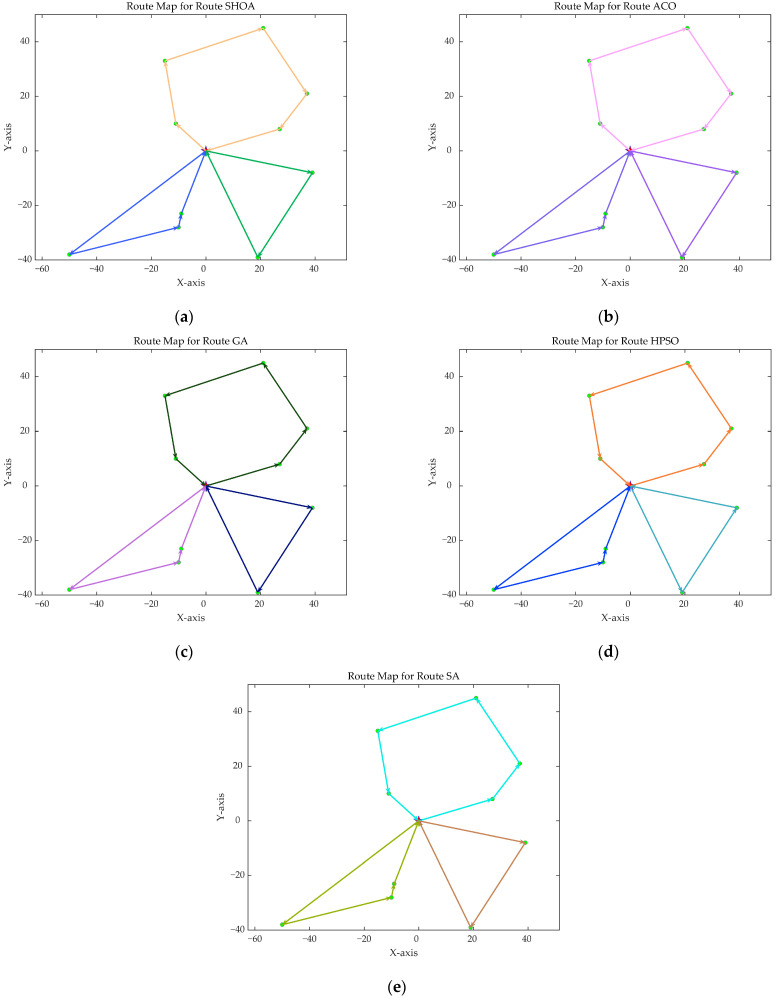
The optimal paths of five algorithms in 10 customer cases. (**a**) SHOA; (**b**) ACO; (**c**) GA; (**d**) HPSO; (**e**) SA.

**Figure 4:**
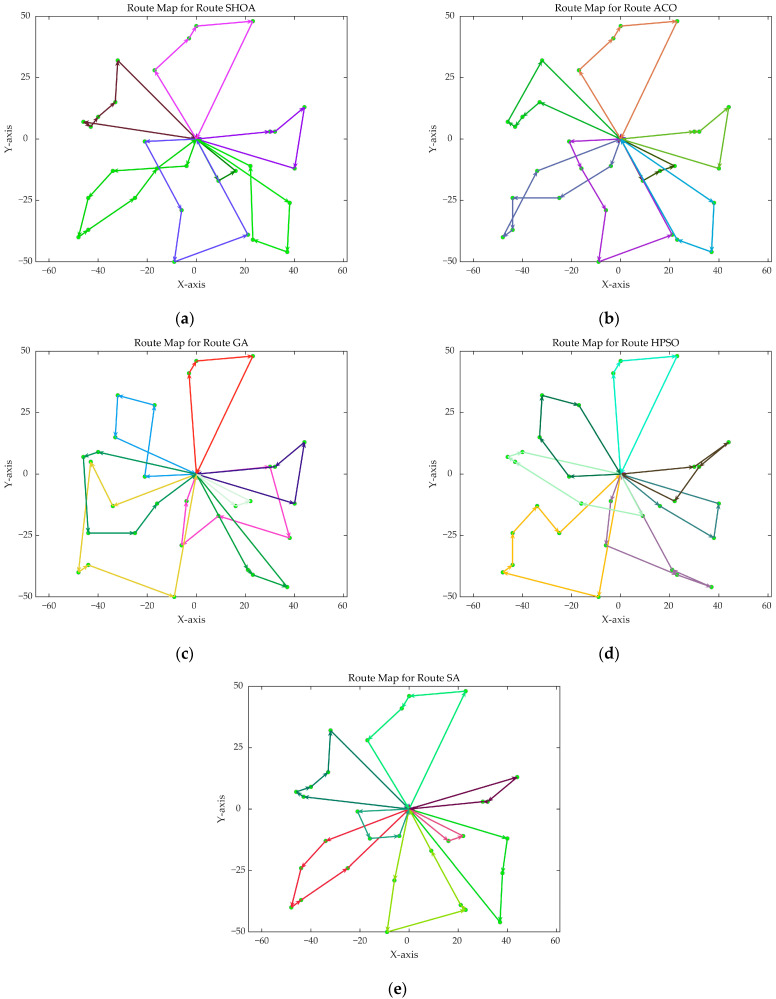
The optimal paths of five algorithms in 30 customer cases. (**a**) SHOA; (**b**) ACO; (**c**) GA; (**d**) HPSO; (**e**) SA.

**Figure 5:**
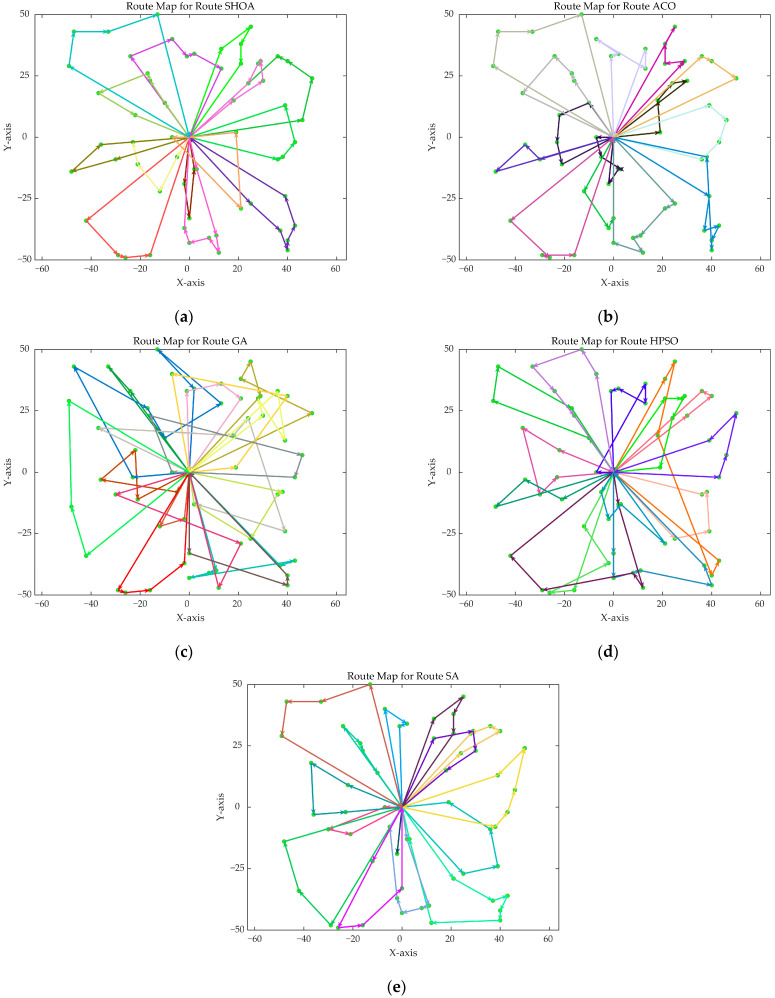
The optimal paths of five algorithms in 60 customer cases. (**a**) SHOA; (**b**) ACO; (**c**) GA; (**d**) HPSO; (**e**) SA.

**Figure 6:**
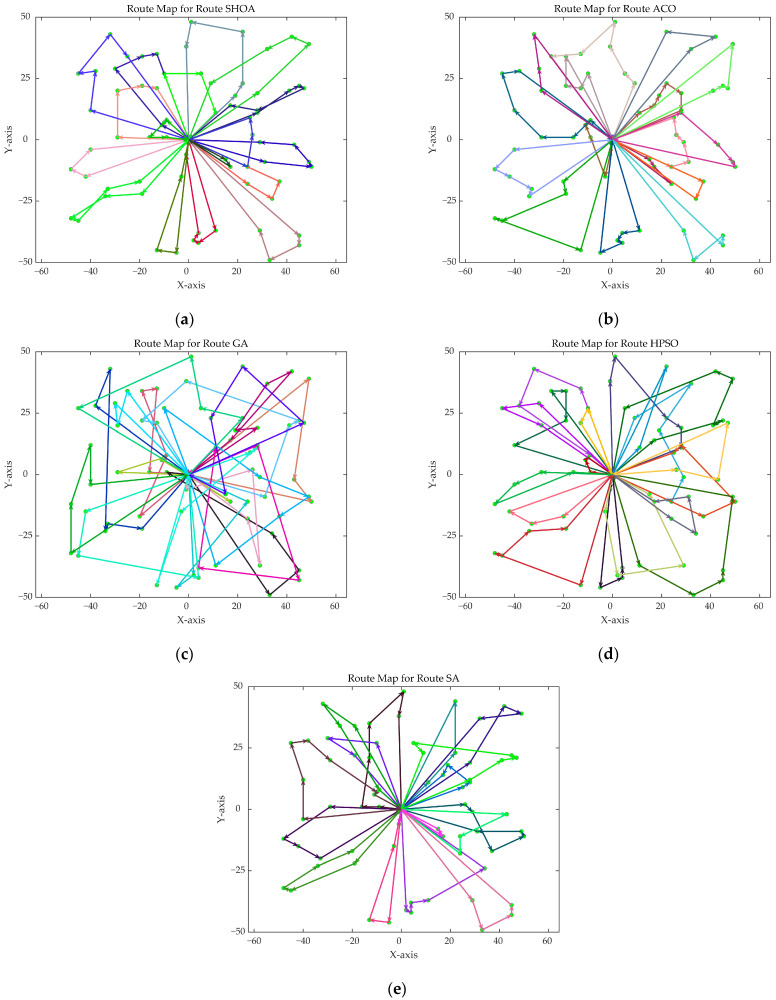
The optimal paths of five algorithms in 70 customer cases. (**a**) SHOA; (**b**) ACO; (**c**) GA; (**d**) HPSO; (**e**) SA.

**Figure 7:**
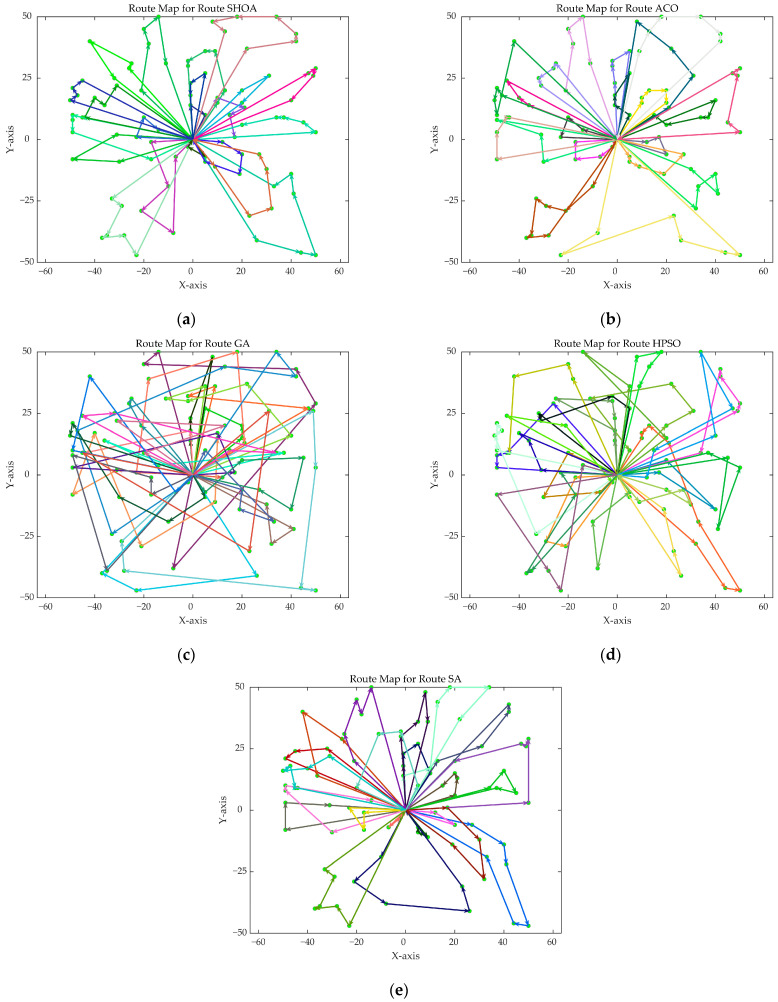
The optimal paths of five algorithms in 90 customer cases. (**a**) SHOA; (**b**) ACO; (**c**) GA; (**d**) HPSO; (**e**) SA.

**Figure 8:**
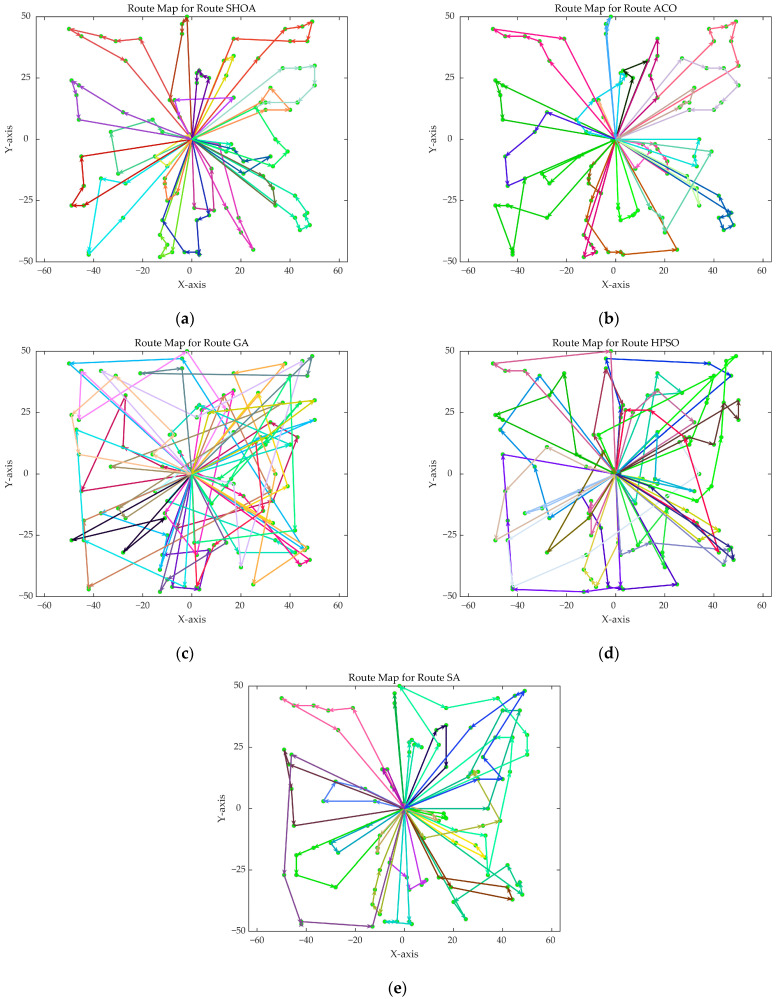
The optimal paths of five algorithms in 100 customer cases. (**a**) SHOA; (**b**) ACO; (**c**) GA; (**d**) HPSO; (**e**) SA.

The population size of all algorithms was 50, and the maximum number of iterations was 500. Figure 2 illustrates the average convergence curves of the SHOA, ACO, GA, HPSO, and SA after 30 runs. For each experimental run, the optimal solution was recorded. Three statistical metrics were computed over 30 independent trials: (1) Best (lowest of optimal values), (2) Mean (arithmetic mean of optimal values), and (3) Std (standard deviation of optimal values). These metrics enabled systematic performance comparison among the SHOA, ACO, GA, HPSO, and SA, as detailed in Table 1. Figure 3, Figure 4, Figure 5, Figure 6, Figure 7 and Figure 8 show the optimal path diagrams of the improved algorithm, ACO, the GA, HPSO, and the SA for each test case.

As shown in Table 1, it is evident that the SHOA demonstrates strong performance in finding optimal solutions across the CVRP instances of varying sizes, with average solutions closely approximating the optimal values and exhibiting high stability. Although the ACO algorithm performs well in small-scale instances, its performance declines in large-scale problems, as reflected in its average solutions and standard deviations, which are inferior to those of the SHOA. In all test cases, the performance of the GA, HPSO, and SA algorithms is consistently inferior to that of the SHOA.

To evaluate the statistical relevance of the computational results, we employed the Wilcoxon rank-sum test—a non-parametric approach for non-normally distributed datasets. The test compares SHOA’s performance metrics against benchmark algorithms under two hypotheses: the null hypothesis (H_0_) of no significant difference versus the alternative (H_1_) of detectable distinction. Using α = 0.05 as the significance threshold, results (Table 2) showed statistically significant differences (*p* < 0.05) between the SHOA’s solution fitness values and all comparator algorithms.

Figure 2 highlights the iteration processes of these algorithms, showing that the SHOA achieves faster convergence and shorter final path lengths, indicating its ability to rapidly identify high-quality solutions while maintaining stability throughout the iteration process. While ACO converges quickly in small-scale instances, its convergence speed and final path length are less competitive in large-scale problems. The GA, HPSO, and SA algorithms exhibit slower convergence and longer final path lengths, suggesting lower stability and efficiency in solving the CVRP. The optimal path diagrams listed in Figure 3, Figure 4, Figure 5, Figure 6, Figure 7 and Figure 8 also show the advantages of the improved algorithm. In summary, the SHOA performs well in solving the CVRP, with high efficiency, accuracy, and stability, making it an excellent choice for such tasks.

### 4.2. Application of the Synergistic Hybrid Optimization Algorithm in the Capacitated Multi-Ship Replenishment Path Planning Problem

This chapter applies the SHOA to address the CMSRPPP, discussing six cases with 20, 40, 60, 80, 100, and 120 replenishment points, respectively. The supply center is located at coordinates [0, 0], while the coordinates of the replenishment points are randomly generated as integer values. The goods reserve quantity at each replenishment point is an integer randomly generated between 100 and 2500, with increments of 100. The risk of encountering a hazard at each point is randomly assigned between 0.1 and 0.4. ACO, the GA, HPSO, and the SA are used to solve these six problems, and the results are compared with those of the SHOA.

**Figure 9:**
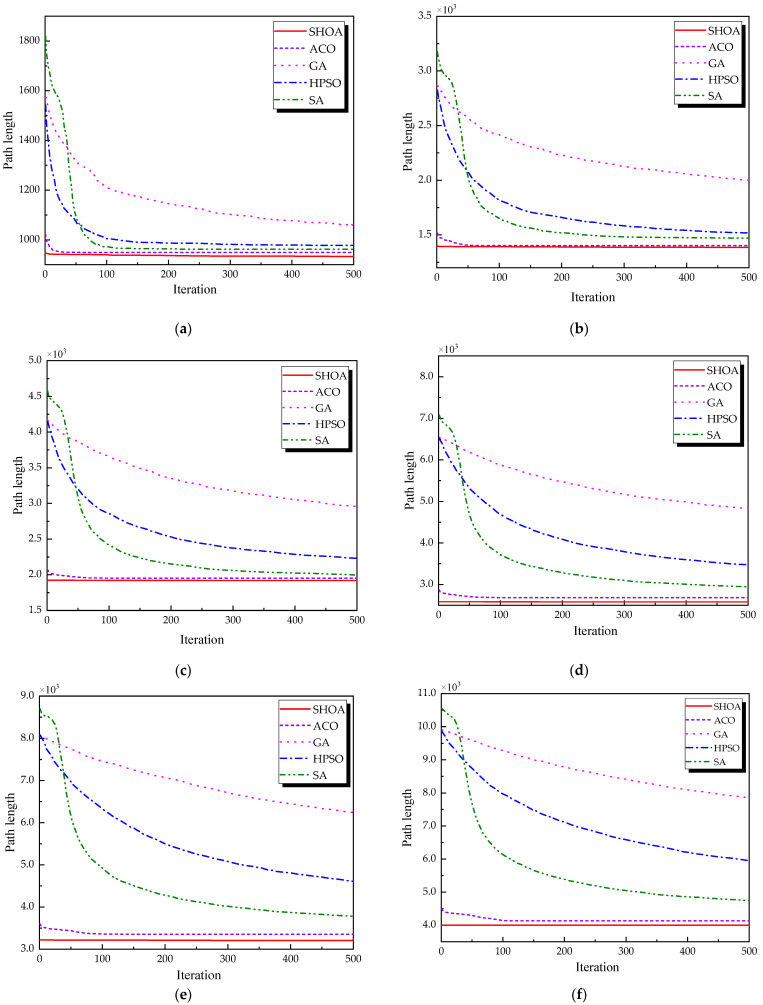
The average convergence curves derived from 30 runs of different algorithms. (**a**) 20 supply points; (**b**) 40 supply points; (**c**) 60 supply points; (**d**) 80 supply points; (**e**) 100 supply points; (**f**) 120 supply points.

**Figure 10:**
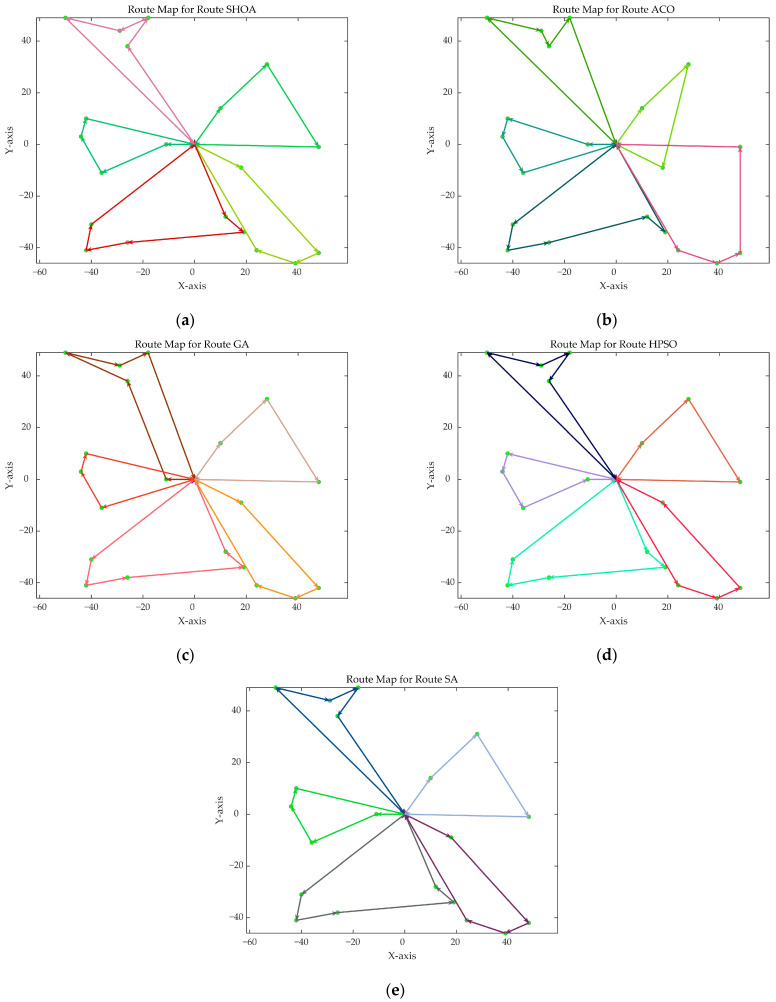
The optimal paths of five algorithms in 20 supply point cases. (**a**) SHOA; (**b**) ACO; (**c**) GA; (**d**) HPSO; (**e**) SA.

**Figure 11:**
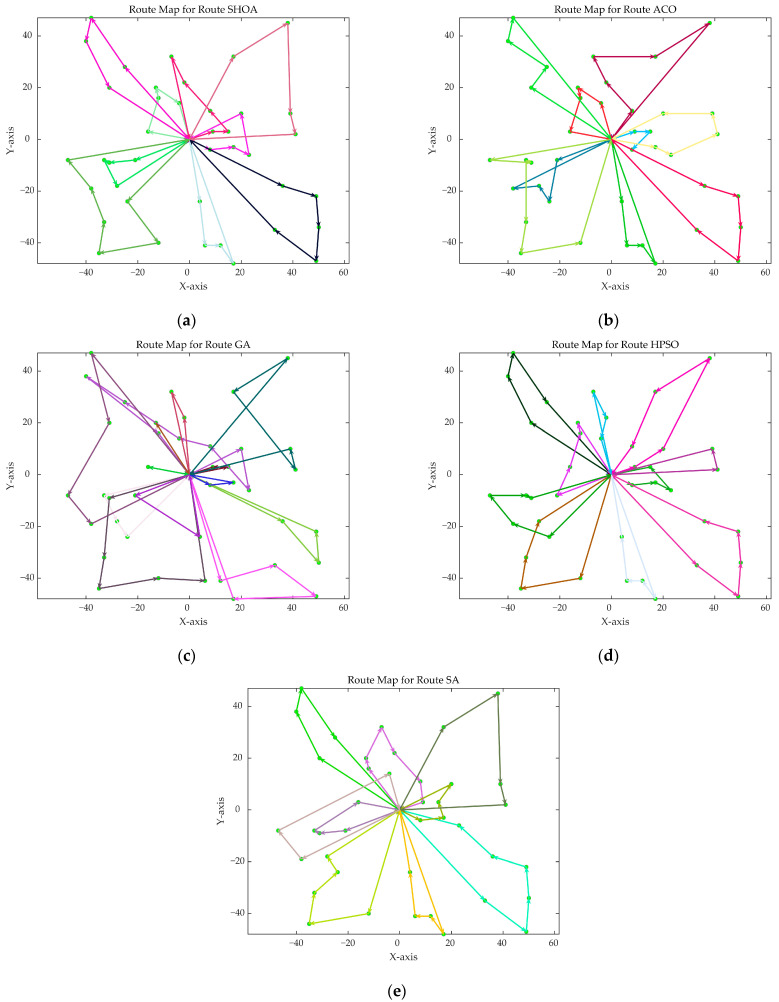
The optimal paths of five algorithms in 40 supply point cases. (**a**) SHOA; (**b**) ACO; (**c**) GA; (**d**) HPSO; (**e**) SA.

**Figure 12:**
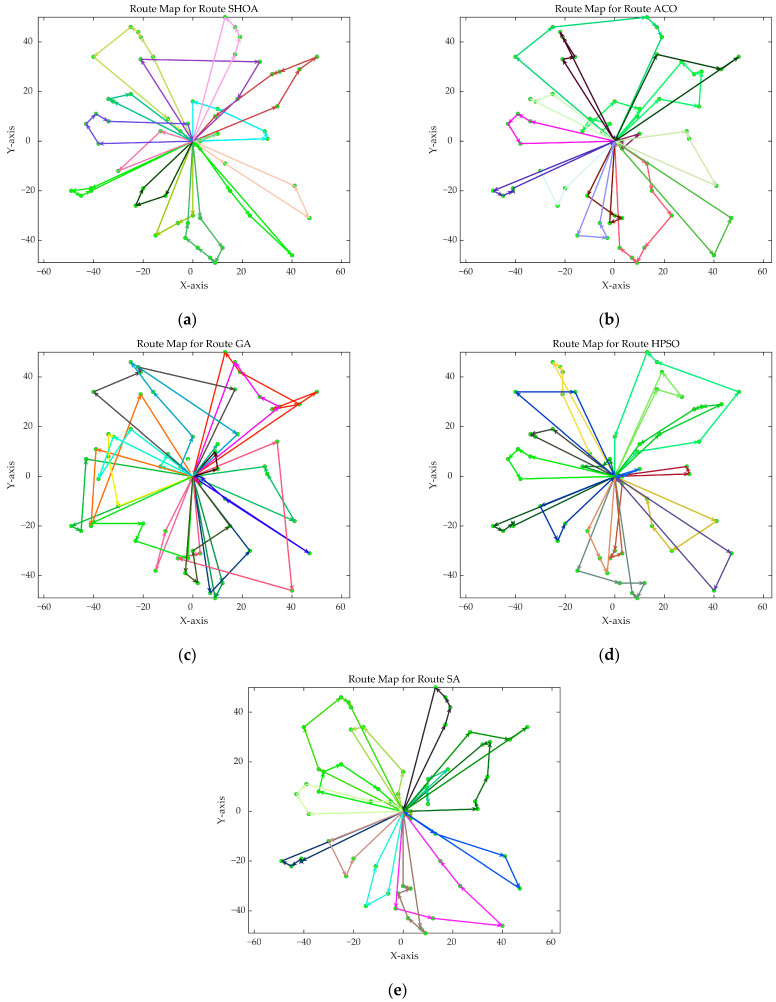
The optimal paths of five algorithms in 60 supply point cases. (**a**) SHOA; (**b**) ACO; (**c**) GA; (**d**) HPSO; (**e**) SA.

**Figure 13:**
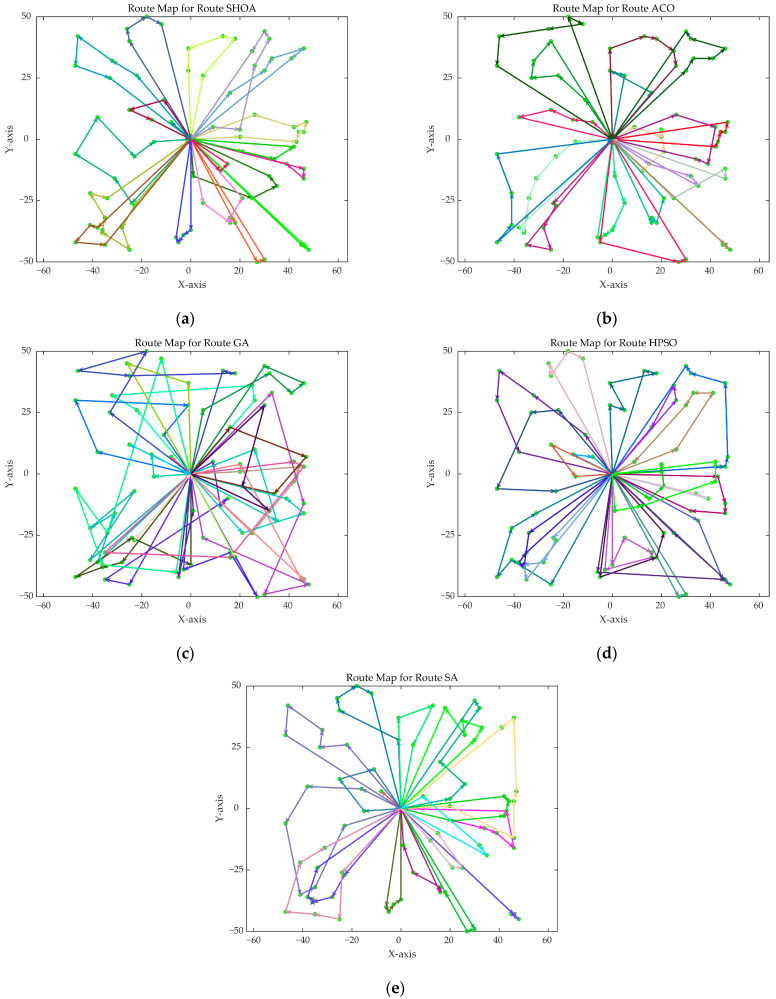
The optimal paths of five algorithms in 80 supply point cases. (**a**) SHOA; (**b**) ACO; (**c**) GA; (**d**) HPSO; (**e**) SA.

**Figure 14:**
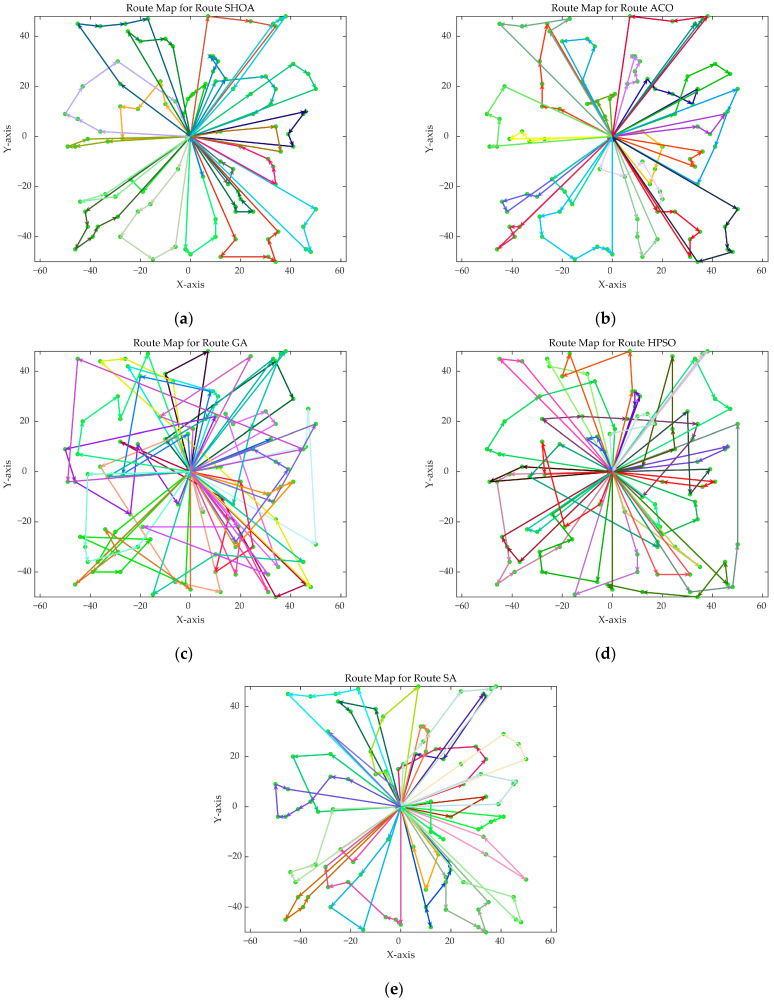
The optimal paths of five algorithms in 100 supply point cases. (**a**) SHOA; (**b**) ACO; (**c**) GA; (**d**) HPSO; (**e**) SA.

**Figure 15:**
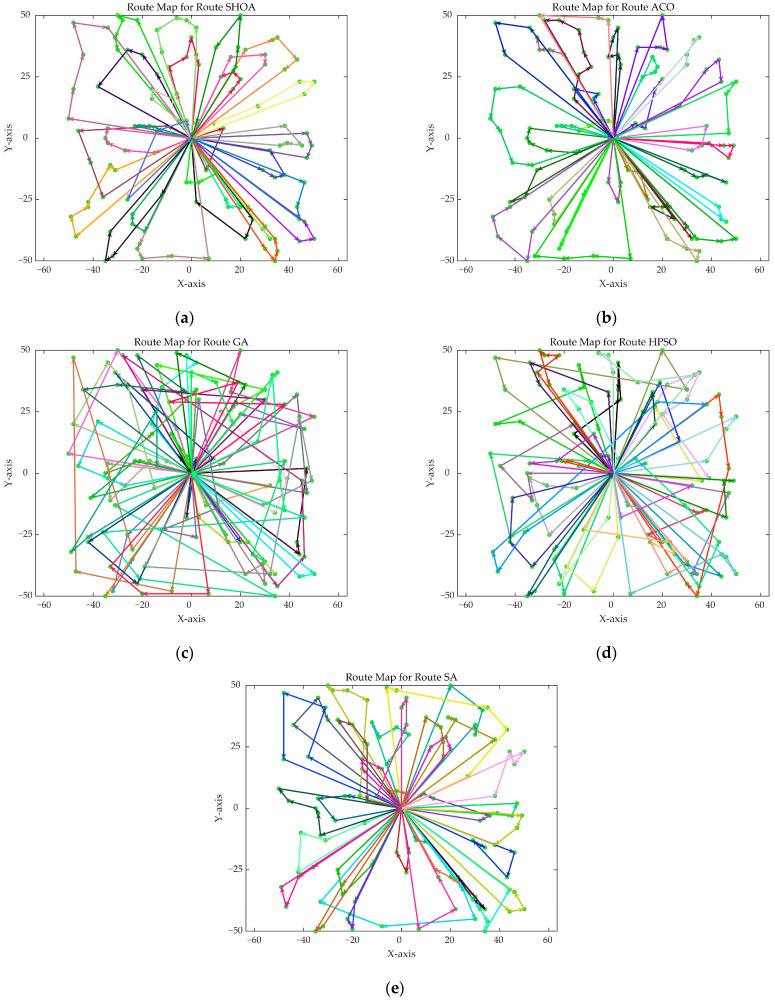
The optimal paths of five algorithms in 120 supply point cases. (**a**) SHOA; (**b**) ACO; (**c**) GA; (**d**) HPSO; (**e**) SA.

Each algorithm operates with a population size of 50 and a maximum of 500 iterations. All algorithms are executed 30 times, and the Best, Mean, and Std of the results are compared and analyzed in Table 3. Figure 9 displays the average convergence curves of all algorithms after 30 runs. Figure 10, Figure 11, Figure 12, Figure 13, Figure 14 and Figure 15 show the optimal path diagrams of the improved algorithm, ACO, GA, HPSO, and SA in each case.

Table 3 indicates that the SHOA’s average solution closely aligns with the optimal solution, exhibiting a minimal standard deviation, which underscores its robust stability. The ACO algorithm performs well in smaller instances but falls short compared to the SHOA in larger ones, both in terms of average solution and standard deviation. The average solution and standard deviation of the GA, HPSO, and SA are inferior to those of the SHOA across all problem scales. Performance differences were evaluated using the Wilcoxon rank-sum test (Table 4), with all test cases showing statistically significant differences (*p* < 0.05) between the SHOA’s fitness values and comparator algorithms.

Figure 9 demonstrates that the SHOA exhibits fast convergence speed and a short final path length during the iteration process, highlighting both its high efficiency and the superior quality of its solutions. The ACO algorithm achieves rapid convergence in small-scale problems, but it does not perform as well as the SHOA in large-scale problems. The GA, HPSO, and SA algorithms exhibit suboptimal performance in both convergence speed and final path length. The optimal path diagrams presented in Figure 10 further illustrate the advantages of the SHOA.

In conclusion, the SHOA demonstrates strong performance in the CMSRPPP, showing significant advantages in solution efficiency, accuracy, and stability, and maintaining stable performance when dealing with problems of different scales.

Integrated above, for simple cases (such as 10 customers in CVRP or 20 supply points in the CMSRPPP), the SHOA and benchmark algorithms generate comparable optimal solutions, though the SHOA demonstrates superior stability. As problem complexity increases, the SHOA maintains leading optimality and robustness. While ACO performs well in small/medium problems, its Best Mean value gap widens significantly in large-scale scenarios. The GA and HPSO show high standard deviations and observable redundant loops in path visualizations. Although the SA occasionally matches SHOA’s optimal solutions, its stability deteriorates markedly. Take large-scale scenarios (e.g., 100 customers in the CVRP and 120 supply points in the CMSRPPP) as an example, the SHOA demonstrated statistically significant improvements over standalone algorithms, reducing average path lengths by 3.2–52.0% and optimal path lengths by 1.5–49.1%, with all cases showing statistical significance (Wilcoxon test *p* < 0.05). This performance advantage was consistently observed across all test scenarios, confirming the SHOA’s superior optimization capability.

## 5. Conclusions

This paper introduces a novel synergistic hybrid optimization algorithm that integrates ACO, CW, and GA to solve the capacitated multi-ship replenishment path planning problem. The study utilizes ACO and CW to generate high-quality initial solutions. Subsequently, the optimal solutions obtained by ACO and the CW, combined with some random solutions to construct the initial population of the GA. This strategy not only enriches the diversity of the population but also provides a broader search space for the genetic algorithm. During the iterative process of the GA, an evolution reverse operation is embedded for local optimization, thereby improving the solution’s quality.

To validate the algorithm’s efficacy, extensive experiments were conducted on both the capacitated vehicle routing problems (CVRP) and CMSRPPP instances. Take large-scale scenarios (e.g., 100 customers in the CVRP and 120 supply points in the CMSRPPP) as an example, the SHOA achieved 3.2–52.0% shorter average path lengths and 1.5–49.1% shorter best path lengths compared to standalone algorithms (ACO, GA, HPSO, SA), with statistically significant improvements (Wilcoxon *p* < 0.05 in 100% of cases). Notably, the SHOA exhibited superior stability, evidenced by minimal standard deviations. The outcomes demonstrate that the algorithm offers substantial advantages over other methods in path planning problem, highlighting its strong potential for practical applications

Nevertheless, there are certain limitations to this research. The study is primarily centered on single-objective static risk issues and does not adequately account for more intricate real-world elements, including multi-item restocking, fluctuations in environmental conditions, and unpredictable demand patterns. Future research should investigate the application potential of the SHOA in multi-objective optimization with varying risk factors to adapt to dynamically changing environments and demands.

## Figures and Tables

**Table 1:** Performance comparison of different algorithms in six CVRP.

Number of Customers	Performance	SHOA	ACO	GA	HPSO	SA
10	Best	403.48	403.48	403.48	403.48	403.48
	Ave	403.48	403.48	415.48	408.53	407.31
	Std	0	2.36e-14	12.59	7.43	6.83
30	Best	845.36	853.51	996.71	874.63	857.36
	Ave	852.02	874.67	1149.36	942.82	905.30
	Std	1.32	10.46	63.19	52.79	34.63
60	Best	1508.10	1539.26	2231.92	1750.03	1580.87
	Ave	1509.60	1581.31	2519.98	1897.81	1649.91
	Std	0.76	16.29	101.28	68.12	40.62
70	Best	1666.49	1694.32	2569.38	2003.97	1730.26
	Ave	1671.10	1743.21	2899.93	2161.69	1826.12
	Std	2.32	14.47	137.25	72.89	57.97
90	Best	1985.74	2037.29	3612.84	2648.74	2164.95
	Ave	1987.81	2061.31	3866.17	2851.45	2308.78
	Std	1.18	12.35	150.61	91.39	67.75
100	Best	2188.36	2221.57	4295.57	3156.49	2520.45
	Ave	2191.22	2309.92	4565.04	3386.99	2674.79
	Std	1.20	23.19	150.43	117.30	79.46

**Table 2:** Wilcoxon rank-sum test (*p*-value) in six CVRP.

Number of Customers	ACO	GA	HPSO	SA
10	0.0214	1.8413 × 10^−10^	6.4301 × 10^−5^	1.2285 × 10^−9^
30	1.6922 × 10^−11^	1.6933 × 10^−11^	1.6933 × 10^−11^	1.6933 × 10^−11^
60	7.7632 × 10^−12^	7.7632 × 10^−12^	7.7632 × 10^−12^	7.7632 × 10^−12^
70	2.9747 × 10^−11^	2.9747 × 10^−11^	2.9747 × 10^−11^	2.9747 × 10^−11^
90	1.5589 × 10^−11^	1.5589 × 10^−11^	1.5589 × 10^−11^	1.5589 × 10^−11^
100	7.8676 × 10^−12^	7.8676 × 10^−12^	7.8676 × 10^−12^	7.8676 × 10^−12^

**Table 3:** Performance comparison of different algorithms in six ship replenishment cases.

Number of Supply Points	Performance	SHOA	ACO	GA	HPSO	SA
20	Best	930.80	936.85	948.21	930.80	930.80
	Ave	933.48	949.18	1059.02	977.70	961.88
	Std	4.99	10.88	44.79	48.72	45.21
40	Best	1349.23	1360.99	1847.91	1407.68	1370.63
	Ave	1385.39	1401.60	1999.32	1519.69	1471.12
	Std	11.87	19.06	93.53	59.28	65.52
60	Best	1909.50	1932.36	2795.60	2077.02	1878.35
	Ave	1919.95	1952.93	2959.96	2230.34	1997.91
	Std	3.81	12.24	110.30	68.54	42.56
80	Best	2567.95	2639.39	4385.85	3145.12	2744.63
	Ave	2579.71	2683.49	4819.11	3477.83	2941.52
	Std	3.27	19.39	206.81	170.18	95.03
100	Best	3191.70	3276.07	5915.68	4315.99	3527.96
	Ave	3205.36	3350.84	6240.00	4607.31	3781.11
	Std	4.87	24.06	170.96	139.42	98.13
120	Best	3988.87	4090.70	7500.33	5639.96	4581.57
	Ave	3998.70	4130.09	7849.41	5949.42	4747.92
	Std	3.51	19.98	191.17	145.36	104.76

**Table 4:** Wilcoxon rank-sum test (*p*-value) in six ship replenishment cases.

Number of Customers	ACO	GA	HPSO	SA
20	1.0959 × 10^−8^	2.3753 × 10^−11^	4.2826 × 10^−8^	3.7353 × 10^−6^
40	2.9792 × 10^−8^	2.9339 × 10^−11^	2.9339 × 10^−11^	3.4600 × 10^−7^
60	2.8773 × 10^−11^	2.8773 × 10^−11^	2.8773 × 10^−11^	5.3421 × 10^−10^
80	1.9482 × 10^−11^	1.9482 × 10^−11^	1.9482 × 10^−11^	1.9482 × 10^−11^
100	1.7836 × 10^−11^	1.7836 × 10^−11^	1.7836 × 10^−11^	1.7836 × 10^−11^
120	1.6179 × 10^−11^	1.6179 × 10^−11^	1.6179 × 10^−11^	1.6179 × 10^−11^

## Data Availability

The data presented in this study are available on request from the corresponding author.
